# Supplementary material for: Risk factors for human infection with mpox among the Mexican population with social security
Source: PLoS One. 2025 Jan 8;20(1):e0313691. doi: 10.1371/journal.pone.0313691 (PMC11709237; doi:10.1371/journal.pone.0313691)
Supplement: S1 Table — (DOCX) [file pone.0313691.s001.docx]

**Table S1. Bivariate analysis of variables associated with laboratory-confirmed mpox cases**

| **Variable** | **Caterogies** | **Sig.** | **Odds ratio (OR)** | **C.I. for OR 95%** | |
| --- | --- | --- | --- | --- | --- |
|  |  |  |  | **Lower** | **Upper** |
| Age group (years) | 15-19 | .370 | 1.577 | .583 | 4.270 |
|  | 20-24 | .008 | 3.371 | 1.367 | 8.315 |
|  | 25-29 | .000 | 7.947 | 3.244 | 19.470 |
|  | 30-34 | .000 | 12.444 | 5.064 | 30.580 |
|  | 35-39 | .000 | 13.592 | 5.473 | 33.757 |
|  | 40-44 | .000 | 12.869 | 5.100 | 32.473 |
|  | 45-49 | .000 | 8.556 | 3.355 | 21.818 |
|  | 50-54 | .002 | 4.480 | 1.704 | 11.778 |
|  | 55-59 | .006 | 4.215 | 1.521 | 11.679 |
|  | 60-64 | .708 | 1.273 | .360 | 4.495 |
|  | 65 + |  | 1.000 |  |  |
| Sex assigned at birth | Male | .000 | 10.875 | 8.277 | 14.289 |
|  | Female |  | 1.000 |  |  |
| Gender | Cisgender women | .000 | 1 |  |  |
|  | Cisgender men | .000 | 9.351 | 7.131 | 12.261 |
|  | Non-binary | .000 | 28.959 | 6.439 | 130.238 |
|  | Unknown/ Not registered | .000 | 4.686 | 3.227 | 6.804 |
| Sexual orientation | Heterosexual | .000 | 1 |  |  |
|  | MSM | .000 | 16.790 | 13.698 | 20.581 |
|  | Bisexual | .000 | 13.046 | 8.268 | 20.584 |
|  | Lesbian | .360 | 2.505 | .351 | 17.860 |
|  | Other | .029 | 6.262 | 1.209 | 32.445 |
|  | Unknown/ Not registered | .000 | 2.151 | 1.549 | 2.986 |
| People living with HIV | Yes | .000 | 8.730 | 7.133 | 10.685 |
|  | No |  | 1.000 |  |  |
| Syphilis | Yes | .000 | 6.082 | 2.613 | 14.154 |
|  | No |  | 1.000 |  |  |
| Hepatitis C | Yes | .004 | 8.203 | 1.943 | 34.628 |
|  | No |  | 1.000 |  |  |
| Hospitalized case | Yes | .473 | 1.131 | 0.808 | 1.582 |
|  | No |  | 1.000 |  |  |
| Fatal case | Yes | .278 | 1.874 | .603 | 5.826 |
|  | No |  | 1.000 |  |  |
| Fever | Yes | .000 | 1.840 | 1.544 | 2.192 |
|  | No |  | 1.000 |  |  |
| Rash (skin/mucosal lesions) | Yes | .708 | 1.286 | .345 | 4.799 |
|  | No |  | 1.000 |  |  |
| - Head | Yes | .763 | 0.976 | .836 | 1.141 |
|  | No |  | 1.000 |  |  |
| - Face | Yes | .012 | 1.216 | 1.043 | 1.418 |
|  | No |  | 1.000 |  |  |
| - Neck | Yes | .000 | 0.757 | .646 | .887 |
|  | No |  | 1.000 |  |  |
| - Oral cavity | Yes | .979 | 0.997 | .814 | 1.222 |
|  | No |  | 1.000 |  |  |
| - Arms | Yes | .000 | 1.504 | 1.274 | 1.775 |
|  | No |  | 1.000 |  |  |
| - Legs | Yes | .909 | 0.991 | .851 | 1.155 |
|  | No |  | 1.000 |  |  |
| - Trunk | Yes | .249 | 1.100 | .935 | 1.295 |
|  | No |  | 1.000 |  |  |
| - Palms/soles | Yes | .924 | 0.992 | .848 | 1.161 |
|  | No |  | 1.000 |  |  |
| - Anogenital region | Yes | .000 | 4.008 | 3.395 | 4.732 |
|  | No |  | 1.000 |  |  |
| Lymphadenopathies | Yes | .000 | 2.393 | 2.049 | 2.795 |
|  | No |  | 1.000 |  |  |
| - L. Cervical | Yes | .039 | 1.188 | 1.009 | 1.398 |
|  | No |  | 1.000 |  |  |
| - L. Axillary | Yes | .360 | 0.875 | 0.656 | 1.165 |
|  | No |  | 1.000 |  |  |
| - L. Inguinal | Yes | .000 | 4.599 | 3.664 | 5.772 |
|  | No |  | 1.000 |  |  |
| Headache | Yes | .100 | 1.146 | 0.974 | 1.349 |
|  | No |  | 1.000 |  |  |
| Articular pain | Yes | .937 | .993 | 0.854 | 1.157 |
|  | No |  | 1.000 |  |  |
| Myalgias | Yes | .282 | 1.091 | 0.931 | 1.279 |
|  | No |  | 1.000 |  |  |
| Cough | Yes | .979 | .997 | 0.814 | 1.222 |
|  | No |  | 1.000 |  |  |
| Nausea | Yes | .209 | .865 | 0.690 | 1.085 |
|  | No |  | 1.000 |  |  |
| Vomit | Yes | .708 | .931 | 0.641 | 1.353 |
|  | No |  | 1.000 |  |  |
| Odynophagia | Yes | .011 | 1.221 | 1.047 | 1.423 |
|  | No |  | 1.000 |  |  |
| Conjunctivitis | Yes | .835 | .968 | 0.710 | 1.320 |
|  | No |  | 1.000 |  |  |
| Lower back pain | Yes | .078 | 1.163 | 0.983 | 1.377 |
|  | No |  | 1.000 |  |  |
| Bleeding ulcers | Yes | .144 | 1.345 | 0.904 | 2.003 |
|  | No |  | 1.000 |  |  |
| Painful ulcers | Yes | .005 | 1.364 | 1.098 | 1.696 |
|  | No |  | 1.000 |  |  |
