# Supplementary material for: Risk factors for human infection with mpox among the Mexican population with social security
Source: PLoS One. 2025 Jan 8;20(1):e0313691. doi: 10.1371/journal.pone.0313691 (PMC11709237; doi:10.1371/journal.pone.0313691)
Supplement: Attachment — Submitted filename: Response to Reviewers.docx [file pone.0313691.s002.docx]

PONE-D-24-01590

Risk factors for human infection with Mpox among the Mexican population.

PLOS ONE

Firstly, all the authors would like to thank you for your time as reviewers, and we have responded to each of your kind inquiries. We believe that thanks to your feedback, this work has improved to achieve the set objectives.

Journal Requirements:

When completing the data availability statement of the submission form, you indicated that you will make your data available on acceptance. We strongly recommend all authors decide on a data sharing plan before acceptance, as the process can be lengthy and hold up publication timelines.

**Answer**: In response to your suggestion, we have placed the available data in an open repository and the link is noted in the manuscript. <https://doi.org/10.6084/m9.figshare.25481266.v1>

Additional Editor Comments:

The Editor's Comments:
**1- Why was the HR caclulated as an epidemiological measure? In your case, the best measure would be the cumulative incidence rate, known as the incidence proportion over the specified	time period.**

**Answer:**

Hazard Ratio (HR) is often calculated as an epidemiological measure because it provides valuable insights into the relative risk or likelihood of an event occurring over time. In many epidemiological studies, particularly those researchers are interested in understanding how certain exposures or interventions affect the risk of developing a particular outcome or disease.

The HR compares the hazard rates, or instantaneous rates of experiencing the event of interest, between different groups. It's commonly used in studies where the time to event data is important, such as in examining the impact of risk factors on disease incidence.

However, when considering the best measure for a specific case, such as the one you mentioned, the cumulative incidence rate, also known as the incidence proportion over a specified time period, might be more appropriate. This measure provides a straightforward understanding of the proportion of individuals who experience the outcome of interest within a given time frame, without considering the time to event aspect.

Therefore, we have removed the Hazard Ratio (HR) from the manuscript.

**2- Given the large sample size, i would suspect that age be normally distributed. Why was the age presented as median (IQR)? I would advise the authors to provide age in different forms (continuous data, categorical data)**

**Answer**:
The variable age, by the Kolmogorov-Smirnov hypothesis test, had a non-normal distribution. (We attach the results).

The interquartile range (IQR) is primarily used in statistics to describe the dispersion of a dataset. The interquartile range is useful in various contexts, especially when the data do not follow a normal distribution.

We have followed your recommendation by presenting additionally the data in a categorical manner.

**Age**

**3- There is lack of information on how the regression analysis was attempted? Which variables were included in the univariate model? and how was the selected of the variables imported in the multivariable model made?**

**Answer**: Thank you for your feedback and inquiry regarding the regression analysis in our study. We understand the importance of transparency in reporting our methods.

In our regression analysis, we initially performed univariate modeling, which involved assessing the relationship between each potential predictor variable and the outcome variable individually. This step allowed us to identify variables that showed significant associations with the outcome. This analysis is available in Supplementary Material 1.

Regarding the selection of variables for the multivariable model, we employed a systematic approach. We considered variables that were not only statistically significant in the univariate analysis but also clinically relevant or theoretically plausible based on existing literature and biological understanding of mpox epidemiology.

**4- Was model fit analysis performed? I known that the authors provided the Hosmer and Lemeshow test results; however, there are NOT consistent with the provided R-squared which reflects poor fit (which indicates that the analyzed factors do not reflect the change in the measured outcomes; there are other factors that were not accounted for). This is also supported by the wide confidence interval for variables that have a large sample size, which would normally reflect a narrow CI (like male in the multivariate model).**

**Answer**: In the new analysis the result of the logistic regression model shows a Nagelkerke R-squared value of approximately 0.485. These values indicate that the model explains around 48.5% of the variability in the dependent variable, respectively. It's important to note that while these values provide a measure of the model's goodness of fit, they may be relatively low, suggesting that there are other variables or factors that could be influencing the dependent variable and are not included in the current model.

Additionally, the model summary shows that the variables "Sex at birth," "Age Group," "Sexual orientation, "Condition" and some signs and symptoms are statistically significant (p < 0.05), indicating that these variables are significantly associated with the dependent variable. The confidence intervals for the regression coefficients also provide information about the accuracy of the model parameter estimates.

In the new analysis we have gained in tightening the confidence intervals of the regression coefficients.

**5- This issue is the most important of all, which is the lack of enough baseline data of included patients. Providing age, gender, sexual orientation, and HIV coinfection is not sufficient. Were not there any other data to be analyzed in your dataset? The interpretation from this dataset, given the scarcity of data, is very limited and not different from that already reported in the literature during the disease outbreak.**

**Answer**: Our data source is the epidemiological surveillance system of mpox in Mexico. We understand that it would be beneficial to have more variables to expand our understanding of this infection. However, we provide valuable information in epidemiological terms, such as the value of cumulative incidence by key groups, which is not documented elsewhere. Additionally, quantifying the strength of association of variables is not universally established in the scientific community. By providing the value of R, we document that these variables only explain the phenomenon by 48%, thus opening a debate about which variables could provide greater insight into this disease.

6**- Was multicollinearity considered? and how was it identified and dealt with?**

**Answer**: Multicollinearity is a statistical phenomenon in which predictor variables in a logistic regression model are highly correlated. For moderate to large sample sizes, the approach to drop one of the correlated variables was established entirely satisfactory to reduce multicollinearity. On the light of different collinearity diagnostics, we may safely conclude that without increasing sample size, the second choice to omit one of the correlated variables, can reduce multicollinearity to a great extent.

Reference: Midi, H., Sarkar, S. K., & Rana, S. (2010). Collinearity diagnostics of binary logistic regression model. Journal of Interdisciplinary Mathematics, [13](https://doi.org/https:/doi.org/10.1080/09720502.2010.10700699)([3](https://doi.org/https:/doi.org/10.1080/09720502.2010.10700699)), 253–267. https://doi.org/10.1080/09720502.2010.10700699

<https://www.tandfonline.com/doi/abs/10.1080/09720502.2010.10700699>

After reviewing the editor's and reviewers' comments, we decided to remove the gender variable, which was clearly correlated with sex.

We also reduced categories in the sexual orientation variable, leaving only the group of men who have sex with men, which has been most strongly associated with mpox infection.

| **Contingency table Sex at birth * Gender** | | | | | | | |
| --- | --- | --- | --- | --- | --- | --- | --- |
|  | | | Gender | | | | Total |
|  |  |  | Female | Male | Non-binary / Bigender / Transgender / Other | Unknown/ Not registered |  |
| Sex assigned at birth | Women | Count | 367 | 2 | 0 | 36 | 405 |
|  |  | % in Gender | 97.9% | 0.1% | 0.0% | 17.2% | 14.3% |
|  | Man | Count | 8 | 2228 | 16 | 173 | 2425 |
|  |  | % in Gender | 2.1% | 99.9% | 100.0% | 82.8% | 85.7% |
| Total | | Count | 375 | 2230 | 16 | 209 | 2830 |
|  |  | % in Gender | 100.0% | 100.0% | 100.0% | 100.0% | 100.0% |

| **Contingency table Sex assigned at birth * Sexual Orientation** | | | | | | | | | |
| --- | --- | --- | --- | --- | --- | --- | --- | --- | --- |
|  | | | Sexual Orientation | | | | | | Total |
|  |  |  | Heterosexual | Gay/HSH | Bisexual | Lesbian | Other | Unknown/ Not registered |  |
| Sex assigned at birth | Women | Count | 353 | 1 | 7 | 3 | 0 | 41 | 405 |
|  |  | % in Sex | 87.2% | 0.2% | 1.7% | 0.7% | 0.0% | 10.1% | 100.0% |
|  | Man | Count | 744 | 1401 | 142 | 1 | 7 | 130 | 2425 |
|  |  | % in Sex | 30.7% | 57.8% | 5.9% | 0.0% | 0.3% | 5.4% | 100.0% |
| Total | | Count | 1097 | 1402 | 149 | 4 | 7 | 171 | 2830 |
|  |  | % in Sex | 38.8% | 49.5% | 5.3% | 0.1% | 0.2% | 6.0% | 100.0% |

**7- In line 203, diabetes was put in the regression mode, where it was not even mentioned in the baseline variables table (Table 1). Were variables selected on purpose? You need to standardize the reporting of your data.**

**Answer:** We have standardized the reporting of information; the diabetes variable has been removed from Table 1 and from the regression model, focusing on variables with biological plausibility and recognized by the scientific community to explain the event of interest.

**8- What comorbid conditions were considered in Table 1? numerical data is required. Was HIV considered a part of these data?**

The epidemiological study contains a question regarding whether the patient has any comorbidity (without specifying which), and it includes specific variables for hepatitis C, syphilis, gonorrhea, and HIV. We agree with you, and in Table 1, specific diseases are described.


Reviewers' comments:

Reviewer's Responses to Questions

**Comments to the Author**

**Reviewer #1: The paper titled "Risk factors for human infection with Mpox among the Mexican population" provides a comprehensive analysis of the Mpox outbreak in Mexico, identifying key risk factors associated with the disease. However, like any scientific study it may have limitations or criticisms.**

**The study is based on data from the Mexican Institute of Social Security (IMSS), which covers 46.3% of the Mexican population. While this is a substantial proportion, findings may not fully represent the entire population, especially those not covered by IMSS, perhaps because of different socioeconomic backgrounds. The results cannot be referred to the entire Mexican population, as the title of the paper states.**

**Answer**: Indeed, it cannot be extrapolated to the Mexican population. Therefore, following your comment, the title and interpretation of the results were adjusted to refer to the population with social security in Mexico.

**The study mentions that some data, such as sexual orientation and living with HIV status, were self-reported. Self-reported data can introduce bias due to underreporting or misreporting, especially if it concerns sexual orientation and HIV status, affecting the accuracy of the associations found.**

**Answer**: Given that this is a study based on epidemiological information and, in general, on medicine, pathological histories are directly queried to the patient and rely on the trust in the doctor-patient relationship to obtain information. However, we acknowledge the information bias you mentioned, so much so that we decided to report it in the methodology. To ensure that the reader takes this observation into account, in the limitations section of the study, we added the following:

This study supplies an overview of mpox infection in the medical-insured population of Mexico. Due to the study's observational nature, we want to clarify that the information comes from a self-reported questionnaire answered by the individuals taking part in the survey, so it is not unbiased, and that the presence of diseases such as gonorrea, syphilis or living with HIV has not been confirmed as a part of the study.

**The study excluded cases without laboratory confirmation. While this strengthens the reliability of confirmed cases, it may also omit cases with clinical symptoms of mpox but lacking laboratory confirmation, potentially underestimating the actual number of cases and the full spectrum of disease presentation.**

**Answer**: You are correct in the observation you make; therefore, we have stated in a paragraph the fact that the incidence of cases may be underestimated.

“This study excluded suspected cases that did not have laboratory results, accounting for 4.3% of the suspected cases. Therefore, the incidence of mpox may be underestimated”.

**Given the observational nature of the study, it can identify associations but cannot establish causality between risk factors and Mpox infection. Longitudinal studies would be necessary to understand the temporal sequence of exposure and infection better**.

**Answer**: We agree that causality cannot be established with this study but does not achieve this objective.

**While the study performed multivariate logistic regression to adjust for several variables, there might be other confounding factors not accounted for, such as socioeconomic status, access to healthcare, and other behavioral factors that could influence the risk of Mpox infection.**

**Answe**r: Acknowledging the validity of the concern raised. Despite the multivariate logistic regression conducted to adjust for various variables, it is true that there may still be other confounding factors not considered, such as socioeconomic status, access to healthcare, and additional behavioral factors that could potentially influence the risk of Mpox infection. These factors indeed play significant roles in epidemiological studies and could impact the interpretation of results. Therefore, it's important to acknowledge these limitations and recognize that further research or adjustments may be necessary to better understand the true relationship between the variables studied and the risk of Mpox infection.

In the discussion, we have acknowledged what you mention as follows: “We acknowledge that the absence of variables such as socioeconomic status, region or place of residence, and sexual behavior, including the number of sexual partners and casual sexual contacts, is a limitation of our study. These factors could potentially influence the risk of mpox infection”.

**The paper briefly mentions the importance of vaccination and epidemiological surveillance but does not delve deeply into specific strategies or challenges related to vaccine distribution, acceptance, and coverage, especially among high-risk groups.**

Answer: the manuscript was complemented as suggested.

**Reviewer #2: It's an important study regarding the outbreak of mpox infections. However, the manuscript could be improved with some additional information:**

Introduction, lines 72-74: it would be more appropraite to report the number of cases and deaths at time of declaring the global health emergency in july 2023 with the most recent update available on the epidemiology of the disease. Also line 80-84, it's more appropriate to report the most recent data

**Answer**: The manuscript was complemented by describing the global epidemiological situation of mpox at the time a PHEIC was declared and with the most recent data published by WHO. The epidemiological situation in Mexico was also complemented with the most recent update.

**Table 1: it would be more appropriate to report the colomn % than row % as it will show the caracteristics of the confirmed cases as compared to cases without mpox, the p-value should remain the same for conclusions on statistical differences. Please also report the min and max age and the mean+sd, and age in relevant categories. what were the symptoms reported, treatments, vaccination status, transmission mode, hospitalisation rates, complications and/or deaths ?**

**Results on cumulative incidence, it would be interesting to show age and sex adjusted rates.**

**Answer**: Thank you for these comments, we made the change of % in columns in table 1 to observe the percentage of each independent variable between the confirmed cases and between the cases without mpox, what indeed do not change the p values.

Regarding other statistical measures of the age variable, because the age variable has a non-normal distribution, in Table 1 we presented the median and the interquartile range, as the most appropriate measures of central tendency and dispersion. The age variable was grouped into categories by five-year periods and is also presented in Table 1.

We include a description of symptoms, hospitalization, and deaths. No data on vaccination status, treatment or complications were collected. Additionally, we included the cumulative incidence by age group and sex.

**Table III: multivariate logistic regression**

**- What was the total sample used**

**Answer:** In the multivariate logistic regression analysis, we utilized the entire sample of cases reported in a total of 2830 patients and it was noted to the manuscript.

**- What was the dependent variable and the size of each group in the analysis**

**Answer:** The dependent variable was the patient's status (confirmed by laboratory as mpox or ruled out), with a total of 1744 patients with confirmed mpox and 1086 patients without mpox.

**- Were there any missing values for some variables.**

**Answer:** Yes, there are missing values in certain variables such as gender, sexual orientation, these cases were considered as 'not registered' and were included in the analysis as missing values.

**- Please show all the crude OR and 95% CI**

**Answer:** We have added in the S1. Supplementary material. Bivariate analysis between studied variables and being a case of MPOX, the crude odds ratios of the analyzed variables.

**- The age would be more appropriate in categories rather than continuous in this context.**

**Answer:** Thank you very much for your observation. Now, age is described by age group.

**- Gender and sexual orientation are likely to have a colinearity in multivariable model, was this tested ?**

**Answer**: We conducted a correlation test between the variables you mentioned: gender and sexual orientation, as well as sex at birth and gender. Since they are strongly correlated, and to avoid collinearity, we decided not to include gender and sexual orientation in the analysis. Instead, we only included gay and men who have sex with men (MSM), considering existing literature. You can observe the analysis performed in the response to the editor (above).

**- What was the reference category for each underlying condition, how many patients had more than one condition ?**

**Answer** In Supplementary Material 2, the frequency of comorbidities and their correlation can be observed. In this analysis, we found that living with HIV positively correlated with living with syphilis and Hepatitis C, and negatively correlated with diabetes.

**- Diabetes seems to be associated with a lower risk of mpox infection, how is this explained

Answer** Thank you for your question. Among the authors, we have carefully considered your inquiry. Firstly, we did not find biological plausibility between diabetes protection and mpox. After correlating living with HIV and diabetes, we believe several conditions may be at play: 1. Diabetes correlates with the age group at a statistically significant level of .254, 2. The incidence of diabetes in Mexico is higher among older age groups. Therefore, we suspect that diabetes is confounded by the age of the patients, and we have decided to exclude it from the multivariate analysis.

| **Correlations** | | | |
| --- | --- | --- | --- |
|  | | Diabetes | Age group |
| Diabetes | Correlación de Pearson | 1 | .254^**^ |
|  | Sig. (bilateral) |  | .000 |
|  | N | 2830 | 2830 |
| Age group | Correlación de Pearson | .254^**^ | 1 |
|  | Sig. (bilateral) | .000 |  |
|  | N | 2830 | 2830 |
| **. La correlación es significativa al nivel 0,01 (bilateral). | | | |

**The conclusion of the manuscript and the abstract is shallow.**

**Answer:** We have developed a more illustrative conclusion of the study, taking into account the reviewers' comments.
