# Supplementary material for: Risk factors for human infection with mpox among the Mexican population with social security
Source: PLoS One. 2025 Jan 8;20(1):e0313691. doi: 10.1371/journal.pone.0313691 (PMC11709237; doi:10.1371/journal.pone.0313691)
Supplement: Attachment — Submitted filename: Response to Reviewers 211024.docx [file pone.0313691.s003.docx]

PONE-D-24-01590R1

Risk factors for human infection with Mpox among the Mexican population with social security.

PLOS ONE

Dear Reviewers, we greatly appreciate your comments. We have addressed each of them.

Reviewer 1.

1. Review the English. There are issues with the language and writing that hinder understanding and compromise the readability and flow.

A=We have carefully reviewed the language and writing in the manuscript.

2. Abstract: The "Materials and Methods" section should include information about the incidence and parameters of the models conducted. The results presented must be supported by the methods.

A= Thank you for your comment, we carefully reviewed the abstract section to ensure it was consistent with the manuscript, including the materials and methods section where we added the cumulative incidence and the models we performed.

3. Consider including more references in the introduction. Each paragraph makes multiple assertions but only cites one reference per paragraph. Why is this the case?

A= The introduction was modified, and several references were included to support the assertions.

4. The introduction needs reorganization. In the first paragraph, the authors start by discussing the 2022 outbreak. In the second and third paragraphs, they describe the transmission mode of the virus, including the hypothesis of sexual transmission relevant to the recent outbreak (2022) but not present in the description of previous cases (before 2022). In the fourth and fifth paragraphs, they discuss the clinical presentation and symptoms. In the sixth paragraph, they revisit (to some extent) the content of the first paragraph. Thus, reorganization is necessary to eliminate repeated sections and improve the coherence of the historical context and background presented.

A= The introduction was reorganized, repeated sections were removed, and background and historical events were arranged chronologically to improve the coherence of the section.

5. It would be worthwhile to include the origin and historical context of mpox, its endemic regions, and how cases were previously described. When discussing the 2022 outbreak, provide temporal context, as well as epidemiological and clinical characteristics that differ from previous cases. It is necessary to contextualize the research problem and the current state of the art for the reader.

A= The origin and historical context of mpox, as well as the description of the epidemiological and clinical features of the multinational outbreak in 2022, were added to the introduction.

6. The last sentence of the final paragraph of the introduction could be moved to the methodology section. The aim at the end of the introduction differs from the aim at the beginning of the methods section. Ensure consistency (consider the abstract as well).
   A= As suggested, the last sentence of the last paragraph of the introduction was moved to the methodology section. The objective was checked to be consistent across all sections such as the end of the introduction, methodology and abstract.
7. At the beginning of the methods section, the authors should briefly explain the health system and the IMSS, including the coverage percentage (currently listed in the last sentence of the introduction). It is not clear whether the IMSS system and the National Epidemiological Surveillance System were linked or if the notifications from NESS are part of IMSS.

A= In the methods section, information was expanded to explain IMSS coverage and the link between the IMSS and the National Epidemiological Surveillance System of Mpox.

8. In the study population section, the authors mention that they included probable and confirmed cases of mpox. Later, they state that they calculated the incidence considering the population covered by IMSS. This is confusing. The study population for each analysis conducted must be clearly defined for the reader.
   A= In the study population section, it is clarified that the study population was all probable cases of mpox reported by IMSS medical units in 2022-2023. In later sections, it is clarified how the cumulative incidence was calculated.
9. The authors do not specify the data access date. Especially for studies using secondary and surveillance data, the access date must be provided as these data are continuously updated by the responsible entities.
   A= In the methodology, at the end of the study population section, it was specified that June 26, 2023 was the date the database was accessed.
10. In the definitions section, a new unnamed category is introduced in the second paragraph, where the authors state that these cases “were also studied for mpox.” What does this mean? In which category do these cases fall?
    A= Thank you very much for your comment. In the variable definition section it was specified that these cases referred to in the commented paragraph are also probable cases of mpox.
11. It is unnecessary to explain sexual orientation definition, just indicate whether it was self-reported (hopefully!) and what categories were included in the study. Gender identity is a separate variable from sexual orientation. The explanation in the methods section is unclear, making it difficult to understand whether these variables were analyzed separately, as they should be.
    A= In accordance with your comments, thank you very much. The definition of sexual orientation was removed. In the methodology section, it was specified that the sexual orientation variable was collected by self-report and the categories of the variable are described. Likewise, the gender variable was collected by self-report and the categories of the variable were described. These variables were analyzed separately.
12. What are confirmatory studies? Are these the tests conducted to confirm mpox cases? If so, this should be defined in the definitions section. For all patients classified as suspected/probable and who provided samples, was PCR conducted for case confirmation?
    A= The subheading for confirmatory studies was removed. The definitions of confirmed case and ruled out case were added to the variable definitions section. It was also clarified that all patients classified as probable cases had samples taken for confirmation by PCR testing performed by authorized laboratories.
13. How were the other variables studied obtained? It is important to describe all variables, including their origin, method of collection (self-reported?), temporal reference, and response categories (when applicable).
    A= In the methodology section, it was described how the other variables such as sociodemographic, epidemiological and clinical variables were collected. The definition of the variables and their categories was also added.
14. Explain how was calculated the incidence. What is the incidence reference? 10,000 people? This needs to be described in the methods.
    A= In the methods section, further information was provided on how the cumulative incidence was calculated.
15. The authors refer to “key populations” without defining what it is and how this variable was created to estimate the stratified incidence.
    A= The term “key populations” was eliminated since, in effect, it was not defined; rather, we refer to population subgroups and this was indicated in the statistical analysis section of the methodology. It was also described how the stratified cumulative incidence was estimated in population subgroups such as MSM and people living with HIV.
16. In the univariate model, what did the authors consider as “strength of association”? (line 184). The described method should be in the main text.
    A= In the univariate model, the term “strength of association” was corrected, clarifying that it is statistical significance.
17. The paragraph (lines 183 to 189) and the following paragraph (lines 190-197) contain repetitions. Review these sections. What parameters were used in the multivariate model? Variables described here appear for the first time in the text without explanations.
    A= Thank you for your comment. We have reviewed the highlighted paragraphs and removed the repeated sections, writing a clearer description of the analysis performed. It was described that the multivariate analysis was performed with the independent variables that showed statistical significance in the univariate analysis, with a level of statistical significance of p<0.05. Additionally, the variables that were indicated in this section were eliminated and included in the variable definitions and data collection section.
18. Ensure consistency of variables presented in the dataset, main text, and tables. For example, the variable “gender” appears under different denominations and different categorizations in the main text, tables and dataset.
    A= Thank you for your valuable comment. We have reviewed the database, the main text, and the tables, and made corrections to the gender and sexual orientation categories. We ensured consistency across the tables, database, and manuscript text.
19. Figure S1 is dispensable. Table S1 needs to be formatted according to the journal's formatting and style guidelines. Adjust the titles.
    A= Thank you very much for your comment. Figure S1 has been removed and Table S1 was formatted according to the PLOS ONE formatting and style guidelines and the title was adjusted.
20. Tables S1 and S2 should be in the same document, in Word/PDF format, following the journal's table formatting rules.
    A= Thank you very much for your comment. Table S1 were inserted into a Word sheet that was converted to a PDF document and Table S2 was removed.
21. Figure 1 (sample composition flowchart) is cited incorrectly at the end of the methods section.

A= We adjusted the citation of Figure 1 according to manuscript body formatting guidelines.

22. In the results, the authors present stratified results by confirmed (by PCR) and unconfirmed cases, which was not described in the methods.
    A= Thank you for your comment. The methodology section was complemented with the description of the comparative analysis carried out to evaluate the clinical symptoms and signs between patients with laboratory-confirmed mpox and those with laboratory-ruled mpox; the statistical test and level of significance used are indicated.
23. In Figure 2, it would be more informative to present the positivity rate per month (% of positives among those tested). In its current form, the figure title is inadequate.
    A= Thank you for your comment, we gladly accept this suggestion and present in Figure 2 the mpox positivity rate per month and change the figure title to a more appropriate one.
24. Only in the description of the results in Table 3 do we learn that the incidence rate presented is per 100,000 IMSS beneficiaries. This needs to be described in the methods and table title.

A= Thank you very much for your comment. We have included in the methodology section the complete description of how the incidence rate per 100,000 IMSS beneficiaries was calculated; we also included this description in the titles and tables where we present the incidence rate.

25. The multivariate model results are presented in a segmented manner. Review this to create a cohesive text with logical flow.
    A= Thank you for your comment, the segmented way of presenting the results of the multivariate analysis has been changed, creating a more fluid text.
26. The first paragraph of the discussion presents an objective conflicting with those in the abstract, the end of the introduction, and the beginning of the methods section. The first paragraph of the discussion should provide an overview of the main study findings, which will be discussed further.
    A= Thank you for your comment, there was indeed a mismatch between the first paragraph of the discussion and the sections indicated, so the first paragraph of the discussion was changed to provide an overview of the main findings of the study.
27. “The discovery of mpox transmission” does not seem an appropriate term.
    A= Thank you very much for your comment, the entire paragraph that included this inappropriate term was removed from the discussion in accordance with the following comment.
28. The second paragraph of the discussion should be in the introduction /repeats content already in the introduction without adding value to the discussion. I suggest removing it.
    A= Thank you for your comment, we agree with your suggestion and removed the paragraph from the discussion as it contained repetitions of the description of the evolution of the multinational mpox outbreak, and effectively did not add value to the discussion.
29. How the authors created the category “bigender", presented at the discussion?
    A= The gender variable was collected according to the categories established in the standardized mpox CRF provided by the national mpox epidemiological surveillance system. The bigender category was one of the categories collected directly from the patient through the questionnaire. Since no patient (0 cases) was reported in this bigender category and based on the review of the articles you recommended to us on the classification of the gender variable, we eliminated this category without affecting the analysis.
30. There seems to be some difficulty on the part of the authors regarding the variables of sex, gender identity, and sexual orientation. I suggest reviewing studies that have clearly addressed these variables. https://www.thelancet.com/journals/lanam/article/PIIS2667-193X(22)00223-X/fulltext
    https://www.thelancet.com/journals/lancet/article/PIIS0140-6736(23)00273-8/fulltext
    A= Thank you very much for your suggestions, we reviewed the recommended articles, and the gender and sexual orientation variables were corrected in the different sections of the manuscript.
31. The authors present results for confirmed cases but refer back to probable cases in the conclusions. The conclusion needs to be revised to address the study's proposed aims.
    A= The discussion and conclusions section has been modified so that it no longer refers to probable cases.
32. All figures and tables need to be reviewed for adherence to formatting standards and appropriate titles.
    A= Thank you for your comment, all tables were reviewed, and the format was adjusted according to the PLOS ONE formatting and style guidelines. Table titles have also been adjusted.

    **Reviwer 2**

    1) Improve the English wording

A= We have carefully reviewed the language and writing in the manuscript.

33. Lack of congruence between the objective of the study, methodology and results. It seems that it is looking for an incidence, but it is not a cohort. We suggest reviewing the methodology when you are looking for description of the characteristics and associated factors, we suggest a cross-sectional study with prevalence and associated factors.

A= Thank you very much for your comment. Since all the cases were new cases identified in the IMSS medical units and reported to the national epidemiological surveillance system, we calculated the cumulative incidence per 100,000 IMSS beneficiaries.

Regarding the type of study, indeed, in the methodology we describe that we conducted a retrospective observational study using a cross-sectional survey to assess mpox cumulative incidence and associated factors.

34. Improve language, avoid the word gay, only MSM, use inclusive language.

A=The language was improved by using inclusive language by eliminating the word gay and using only the term MSM throughout the manuscript.

35. Table 1.- two columns are not necessary.

A= Based on your comment, the second column of Table 1 was removed.

36. The results are reported in means with IQR, but due to the size of the sample it is worth looking at its distribution.

A= The variable age, by the Kolmogorov-Smirnov hypothesis test, had a non-normal distribution. (We attach the results).

The interquartile range (IQR) is primarily used in statistics to describe the dispersion of a dataset. The interquartile range is useful in various contexts, especially when the data do not follow a normal distribution.

**Age**

37. The OR data are poorly written, it should be as follows: MSM OR 8.28 (95%CI 6.50 – 10.55), p<0.001; the semicolon is used to separate ideas

A=We have revised the data presentation as suggested, including the appropriate use of the semicolon to separate the ideas and ensuring that each odds ratio (OR) is followed by its corresponding confidence interval (95% CI) and p-value.

38. 207-208 eliminate, emphasize the main results of your research

A= Thank you for your comment, the highlighted lines have been removed to emphasize the main results.

39. 210-211 Seems like information is repeated. 212 The terminology “American House” is not well understood. 215 Do we have the complete data to affirm Mexico is the fourth country in America with Mpox cases?

A= Thank you for your comment, the paragraph that includes the highlighted lines was deleted, since it effectively contained repeated information from the description of the multinational outbreak of mpox and inappropriate terms, so in response to previous comments, we deleted it because it did not add value to the discussion.

40. Discussion could improve if the author compares local data with other similar studies in other countries. 223: We suggest that cumulative incidence could not be reported with de current methodology

A= Thank you for your comments. The discussion was improved by comparing the results with other similar studies in other countries. In addition, the methodology section clearly described how cumulative incidence was calculated.

41. Idea of 225-226 is not understood

A= Thank you very much, we have modified the wording to better understand the idea.

42. The conclusion does not answer the main objective.

A= The discussion and conclusions section has been modified in order to improve the understanding of the study, ensuring consistency between the different sections.

43. It is difficult to consider a descriptive study

A= Thank you for your comment. We have changed the description of the type of study we conducted in the methodology section.

The authors.
